# Seeing is Believing: Inclusion of Biomedical Scientist Educators as Observers on Clinical Rounds

**DOI:** 10.1007/s40670-022-01546-5

**Published:** 2022-04-18

**Authors:** Alison Clay, Matt Velkey, Kathryn M. Andolsek, Nancy W. Knudsen

**Affiliations:** 1 Associate Adjunct Professor of the Practice in the Department of Medical Education, Duke University, School of Medicine, Durham, NC USA; 2 Department of Cell Biology, School of Medicine, Duke University, Durham, NC USA; 3 Department of Family Medicine and Community Health, School of Medicine, Duke University, Durham, NC USA; 4 Department of Anesthesiology, School of Medicine, Duke University, Durham, NC USA

**Keywords:** Basic science, Preclinical, Rounds, Undergraduate medical education

## Abstract

Increasingly, medical school curricula seek to integrate the biomedical and clinical sciences. Inclusion of the basic sciences into the clinical curricula is less robust than including clinical content early in medical school. We describe inclusion of biomedical scientists on patient care rounds to increase the visibility of biomedical sciences, to nurture relationships between clinicians and biomedical scientists, and to identify additional opportunities for integration throughout medical school.

The content, duration, and role of biomedical science educators (BMSE) in the preclinical years of medical school have been debated for years [1]. Increasingly, medical school curricula seek better integration of the basic and clinical sciences, and the Association of American Medical Colleges (AAMC) holds allopathic medical schools responsible for this integration [2, 3]. However, biomedical scientist educators may not be prepared for integrating clinical content [4]. Medical schools have described many methods for trying to include basic science content in the clerkships, from breaks in the clerkship to revisiting basic science content to case-based learning and new assessment tools [5]. We describe inclusion of BMSE into patient care and hospital-based rounds to allow scientists to explore students’ clinical education as well as to foster relationships between clinical and preclinical faculty and identify opportunities for basic science correlations during clinical encounters and clinical correlations in the preclinical curriculum.

Preclinical course directors and postdoctoral fellows who taught preclinical basic science courses were invited to participate (Fig. 1). The electronic invitation asked BMSE about availability, interest in specific disciplines, and if they wanted a monogrammed white coat with their academic credentials. BMSE received an orientation that included common rounding practices, infection control policies, and scripted language for use as an introduction to the patient and team. BMSE considered how often scientific content was or could be included in rounds. An optional observation “checklist” derived from data on peer observations of clinical teaching was provided [6].

**Fig. 1:**
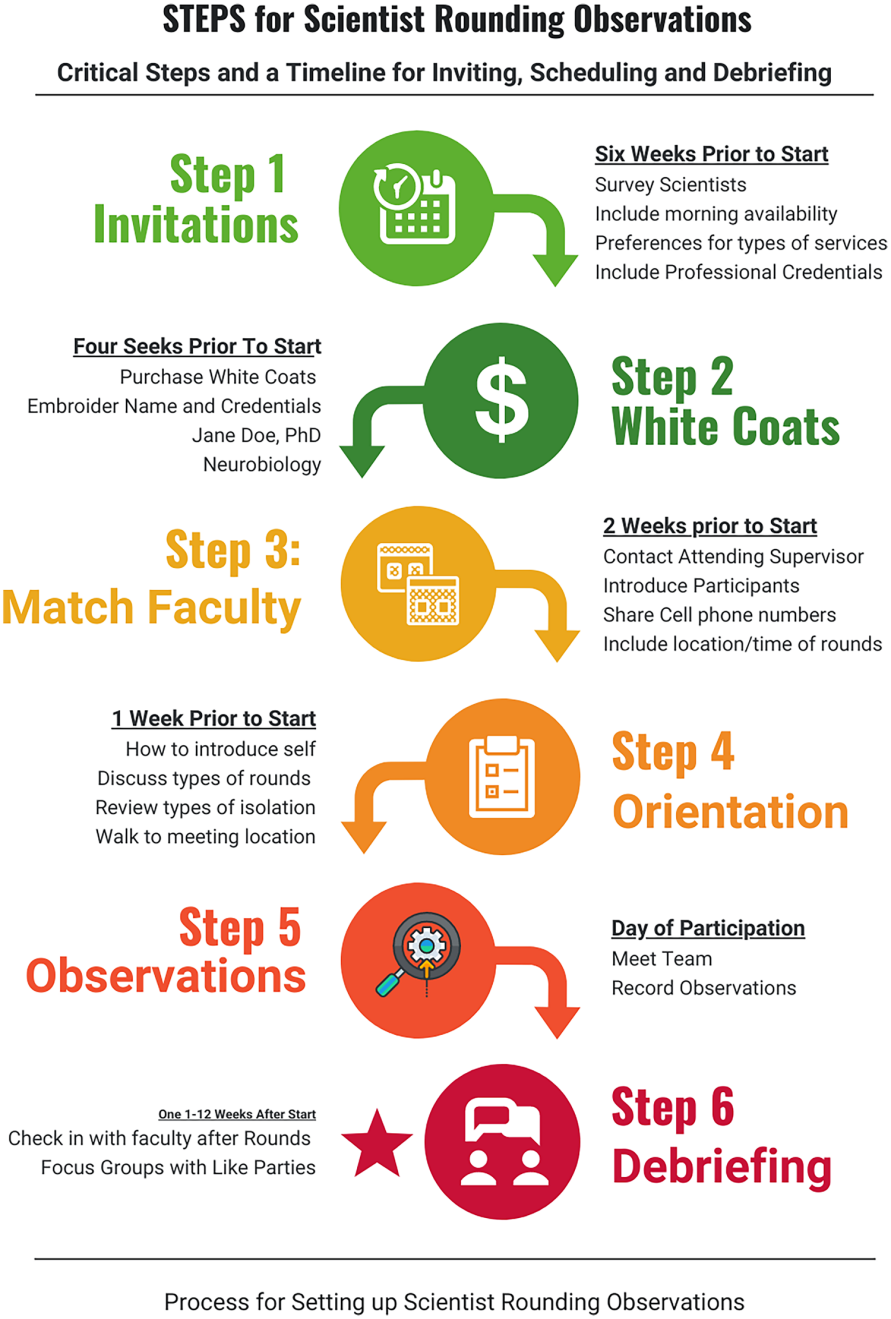
Key steps for BMSE rounding observations

Twelve BMSEs (1 EdD and 11 PhDs) participated in 26 rounding observations on eight different services (medicine, surgery, etc.) with 17 clinicians. Individuals had PhDs in education, cell and developmental biology, biochemistry, neural biology, biology, and evolutionary biology. The program was sustainable: only one BMSE elected not to participate the following semester because he/she felt it was a violation of privacy to witness difficult conversations. Three BMSE requested additional sessions the same semester. All clinician participants agreed to host BMSE in the future. As a direct result of this effort, a new team-based learning exercise with a MD-PHD pair was developed for first year preclinical education. In the clinical departments, one BMSE was invited to contribute as an expert in regularly scheduled multidisciplinary conferences.

BMSEs and clinicians had the opportunity to debrief separately during an informal focus group each semester. Themes from focus group questions were recorded and shared in their entirety here.

BMSE shared several aspects of rounds that surprised them. Universally, on services of their choice, BSME observed content related to their teaching. Educators also noted that students spent time on activities that were not commensurate to time devoted to that content in the preclinical curriculum. Examples included the electronic health record, logistics of efficient patient care, and social determinants of health. One faculty member estimated that > 50% of rounds and student activities included health system science and social sciences, saying, “They covered content I taught, but more surprising was what they did that I didn’t teach.” Another revelation was the stark contrast of assessment in the preclinical years when compared to rounds. Faculty questioned how supervisors assess students’ clinical skills on rounds, particularly when a whole team is contributing to the discussion of the patient. For example, how do you assess a student’s ability to develop a management plan, when the whole team is developing that plan together?

The focus group also identified a wide range of opinions towards the role of BMSEs during rounds in the future. All but one clinician felt BMSE should actively participate on rounds. One clinician asked educators to provide a connection between the basic sciences and content from rounds that day. However, scientists were divided on whether they wanted to be asked this question without knowing the topic in advance.

BMSE also shared aspects of this project they valued. Educators appreciated the personalized white coats and felt more secure in the clinical environment when their credentials were visible to the team. The group appreciated the 1:1 orientation and felt prepared to meet the team. Rounding improved educators’ understanding of the student experience transitioning to the clinical year (for example, where to stand on rounds, when to contribute, potentially being unprepared, and uncertainty about evaluation). Educators enjoyed the opportunity to see their former students “in action” and witness their growth as professionals, often more than a year removed from previous interactions. Students appreciated seeing their preclinical faculty in the clinical setting.

We successfully introduced BMSEs as team members on patient care rounds. This integration occurred at the center of, not separate to, patient care activities. As a result, BSME felt greater awareness of the expectations of clinicians towards medical students. New educational collaborations between clinicians and faculty formed. Success of this program included centrally scheduling the activity through the School of Medicine, offering a variety of clinical experiences, connecting faculty directly to one another and 1:1 orientation.

## Data Availability

The datasets generated and/or analyzed during the current study are available from the corresponding author on reasonable request.
